# Aquatic Peptide: The Potential Anti-Cancer and Anti-Microbial Activity of GE18 Derived from Pathogenic Fungus *Aphanomyces invadans*

**DOI:** 10.3390/molecules28186746

**Published:** 2023-09-21

**Authors:** Manikandan Velayutham, P. Snega Priya, Purabi Sarkar, Raghul Murugan, Bader O. Almutairi, Selvaraj Arokiyaraj, Zulhisyam Abdul Kari, Guillermo Tellez-Isaias, Ajay Guru, Jesu Arockiaraj

**Affiliations:** 1 Department of Medical Biotechnology and Integrative Physiology, Institute of Biotechnology, Saveetha School of Engineering, Saveetha Institute of Medical and Technical Sciences, Thandalam, Chennai 602105, Tamil Nadu, India; 2 Toxicology and Pharmacology Laboratory, Department of Biotechnology, Faculty of Science and Humanities, SRM Institute of Science and Technology, Chengalpattu District, Kattankulathur 603203, Tamil Nadu, India; 3 Department of Molecular Medicine, School of Allied Healthcare and Sciences, Jain Deemed-to-be University, Whitefield, Bangalore 560066, Karnataka, India; 4 Department of Zoology, College of Science, King Saud University, P.O. Box 2455, Riyadh 11451, Saudi Arabia; 5 Department of Food Science & Biotechnology, Sejong University, Seoul 05006, Republic of Korea; 6 Department of Agricultural Sciences, Faculty of Agro-Based Industry, Universiti Malaysia Kelantan, Jeli Campus, Jeli 17600, Malaysia; 7 Advanced Livestock and Aquaculture Research Group, Faculty of Agro-Based Industry, Universiti Malaysia Kelantan, Jeli Campus, Jeli 17600, Malaysia; 8 Department of Poultry Science, University of Arkansas, Fayetteville, AR 72701, USA; 9 Department of Cariology, Saveetha Dental College and Hospitals, Saveetha Institute of Medical and Technical Sciences, Saveetha University, Chennai, 600077, Tamil Nadu, India; ajayguru.sdc@saveetha.com

**Keywords:** subtilisin-like peptidase, virulent factor, anti-cancer molecule, anti-microbial mechanism, oxidative stress

## Abstract

Small molecules as well as peptide-based therapeutic approaches have attracted global interest due to their lower or no toxicity in nature, and their potential in addressing several health complications including immune diseases, cardiovascular diseases, metabolic disorders, osteoporosis and cancer. This study proposed a peptide, GE18 of subtilisin-like peptidase from the virulence factor of aquatic pathogenic fungus *Aphanomyces invadans*, which elicits anti-cancer and anti-microbial activities. To understand the potential GE18 peptide-induced biological effects, an in silico analysis, in vitro (L6 cells) and in vivo toxicity assays (using zebrafish embryo), in vitro anti-cancer assays and anti-microbial assays were performed. The outcomes of the in silico analyses demonstrated that the GE18 peptide has potent anti-cancer and anti-microbial activities. GE18 is non-toxic to in vitro non-cancerous cells and in vivo zebrafish larvae. However, the peptide showed significant anti-cancer properties against MCF-7 cells with an IC50 value of 35.34 µM, at 24 h. Besides the anti-proliferative effect on cancer cells, the peptide exposure does promote the ROS concentration, mitochondrial membrane potential and the subsequent upregulation of anti-cancer genes. On the other hand, GE18 elicits significant anti-microbial activity against *P. aeruginosa*, wherein GE18 significantly inhibits bacterial biofilm formation. Since the peptide has positively charged amino acid residues, it targets the cell membrane, as is evident in the FESEM analysis. Based on these outcomes, it is possible that the GE18 peptide is a significant anti-cancer and anti-microbial molecule.

## 1. Introduction

A peptide-based drug development approach has recently gained global research attention. Peptide or peptide-based drugs are widely used to address several health complications, such as immune diseases, cardiovascular diseases, metabolic disorders, osteoporosis and cancer [1]. About eighty peptide-based drugs with therapeutic effects are commercially available, one hundred and fifty peptides are in the clinical evaluation phase and about six hundred are in the preclinical trial phase [2]. Anti-cancer peptides (ACP) specifically target the cancer cells through their ability to cross the cellular membrane and adhere to the cancer cell receptors [3]. As per the World Health Organization (WHO) report, cancer is the second major cause of death before age 70 in 183 countries. Cancer is the leading cause of mortality and is a substantial hurdle in improving the global survival rates [4,5]. According to the Cancer Research Center, in the United Kingdom, the rate of the occurrence of cancer will grow up to 62% by 2040 [6]. Breast cancer is the most frequently diagnosed cancer in women. It affects one in every four women; cancer deaths have increased to 11.7% of all other cancer cases [5].

Anti-microbial peptides (AMPs) are short peptides that play a vital role in promoting immune responses and protecting organisms from a wide range of pathogenic microbes [7]. Mostly, both ACPs and AMPs have common characteristics, as discussed by Hoskin and Ramamoorthy [8]. ACPs preferentially target cancer cells and mediate the electrostatic interactions with cancer cell membrane receptors [9]. Similarly, AMPs mostly target pathogenic microbes; bacteria have negatively charged membranes, which promote the first electrostatic interaction of AMPs. While all ACPs are not AMPs, both classes of peptides have common biological functions [10]. Proteins and peptides, derived from a pathogenic fungal origin, have significant anti-cancer and anti-microbial activities. A recent report showed that in *Aspergillus fumigatus*, the pathogenic fungal-derived virulence factor has small molecules (SMs), named fumitremorgins, with significant anti-cancer activity [11].

Recent studies have reported on the (i) transcriptomic analysis of the innate immune response of the snakehead murrel fish (*Channa striatus*) against the aquatic pathogenic fungus (*Aphanomyces invadans*)*,* which causes epizootic ulcerative syndrome [12]; and (ii) the transcriptome analysis of the *Aphanomyces invadans* to understand the list of virulence factors responsible for the infection. Nine proteins have been identified from the transcriptome of *A. invadans,* which are potential virulence factors based on the various protein domains such as toxic, hydrolytic and membrane lytic enzymes [12]. Based on these reports, it is only logical to hypothesize that these identified virulence factor-derived peptides may possess diverse biological functions. In support of our proposition, the MP12 peptide, which was derived from the virulence factor, cysteine-rich trypsin inhibitor protein, showed anti-cancer activity against human laryngeal cancer [13]. Therefore, to evaluate our postulation on the peptides derived from virulence factors, this study has established the bio-functional properties of a peptide, GE18, derived from the virulent protein.

## 2. Results

### 2.1. In Silico Investigation of the Protein and GE18 Peptide

The ExPASy translate tool results showed that 641 amino acids encoded from the full-length cDNA sequence have been selected from the transcriptomic data. The amino acid sequence showed 100% identity with the hypothetical protein H310_13544 of *Aphanomyces invadans,* (NCBI Reference Sequence: XP_008879315.1) on the BLAST analysis. In the analysis, protein domains, such as serine proteases and subtilase, were observed. Hence, this specific sequence has been named a subtilisin-like peptidase protein. The physicochemical properties of the protein have been summarized in Table S1. The 3D structural view of the protein is represented in Figure S1A.

The identified peptide, GE18, had 18 amino acids: NH2 terminal—Gly—Ala—Gly—Ile—Val—Val—Ala—Ser—Ile—Asp—Thr—Gly—Val—Arg—Val—Ser—His—Glu—COOH terminal. The HeliQuest analysis of the GE18 peptide showed the net charge at pH 7:−1, hydrophobicity:0.388, Polar residues + GLY:10, non-polar residues:8, hydrophobic moment:0.235, uncharged residues + GLY:7 (HIS 1, SER 2, THR 1, GLY 3), charged residues:3 (ARG 1, GLU 1, ASP 1), the GE18 peptide was predicted to have a non-toxic score value of 8.446198 × 10^−23^ on ToxIBTL prediction and the other properties of GE18 peptide are listed in Table S1. The 3D structure of the peptide and its helical wheel representation has been shown in Figure S1B,C.

### 2.2. Peptide Toxicity Analysis

#### 2.2.1. Effect of Peptide Toxicity on L6 Cells

The toxic effect of the GE18 peptide was investigated using both models, in vitro (L6 cells) and in vivo (zebrafish embryo). The in vitro toxicity results demonstrated that the GE18 peptide-mediated cell viability was 91.73 ± 2% at 10 µM, 90.65 ± 2.9% at 20 µM, 87.91 ± 3.05% at 30 µM, 87.29 ± 1.9% at 40 µM and 84.44 ± 3.8% at 50 µM. Furthermore, the untreated control showed 100% cell viability, and the positive control (0.01% Triton x-100 challenge) showed 20.44 ± 3.5% cell viability. The positive control group showed a significant decrease in cell viability (*p* < 0.05). These observations reveal that the peptide was non-toxic to L6 cells, as shown in Figure S2.

#### 2.2.2. Effect of Peptide Toxicity on Zebrafish Larvae

An in vivo toxicity assay has been performed by using the 24 to 96 hpf zebrafish larvae. The hatching rate was calculated at 48 hpf. The peptide treatment group showed a similar hatching rate compared with the untreated control, whereas the positive control (1 mM H_2_O_2_) showed a significant decrease in the hatching rate of 23.25 ± 1.58% (Figure S3A). At 72 hpf, the heart rate of the peptide-treated larvae showed a slight variation in the higher peptide concentrations: 40 µM had 171 ± 2.1, and 50 µM had 172 ± 1.3 heartbeat/min compared with the untreated control group. The positive control group showed a substantial reduction in the heart rate: 126 ± 2.3 heartbeat/min (Figure S3B).

The mortality and survival rates at 96 hpf are shown in Figure S3C. The mortality rate of the peptide exposure group was 11.23 ± 1.23% at 10 µM, 10.56 ± 1% at 20 µM, 12.78 ± 1% at 30 µM, 19.75 ± 1.5% at 40 µM and 20.46 ± 1.8% at 50 µM. Mortality in the positive control group was 82.31 ± 1.32% when compared with the untreated control (Figure S3D). The peptide treatment group’s survival rate was 92.58 ± 1.59% at 10 µM, 89.45 ± 0.98% at 20 µM, 85.26 ± 1.6% at 30 µM, 83.21 ± 1.65% at 40 µM and 81.25 ± 1.57% at 50 µM. The positive control group showed a substantial reduction in the survival rate: 21 ± 1.58% compared to the control group. These observations reveal that the peptide exposure did not affect the mortality and survival rates of the zebrafish larvae.

**Figure 1:**
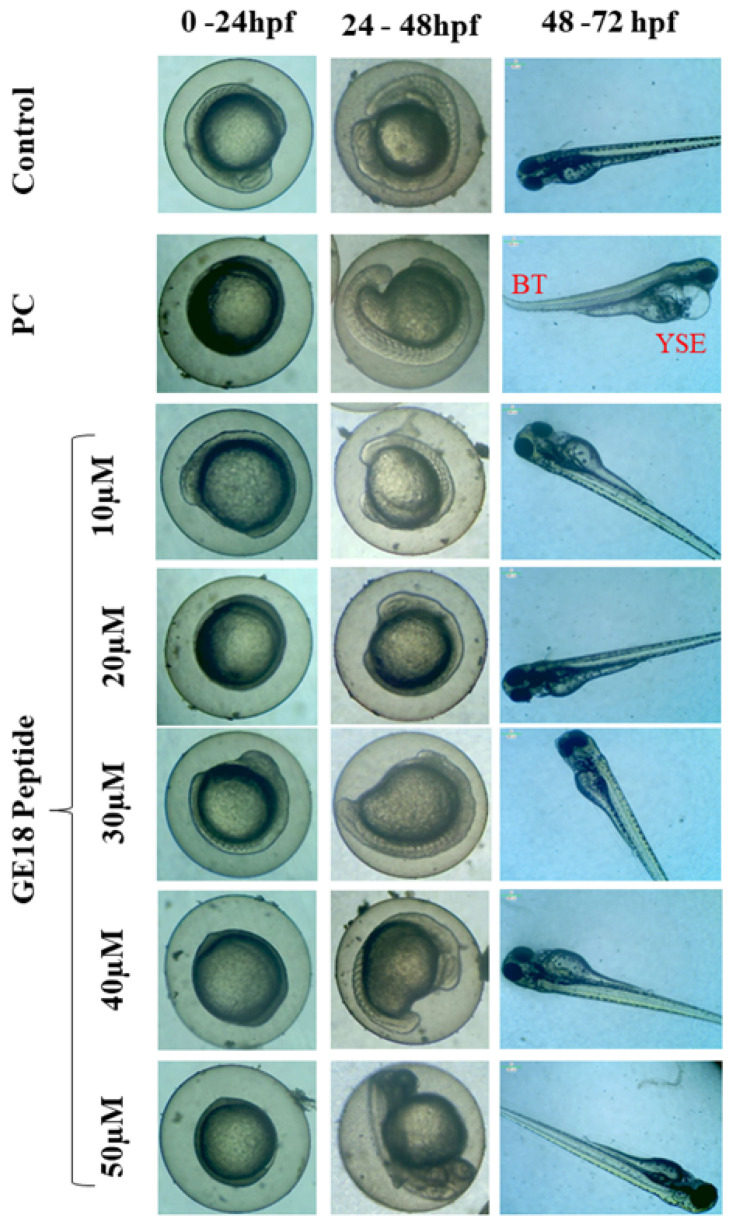
Developmental toxicity on zebrafish larvae’s 0 hpf to 72 hpf. The larva was treated with five different peptide concentrations, control (untreated group) and positive control (PC), which were 1 mM H_2_O_2_. PC showed abnormal morphologies such as yolk sac edema (YSE) and bent tail (BT).

During 0 to 96 hpf, there were peptide-induced morphological changes which were observed under microscopic examination. There were no abnormalities in the morphology of the zebrafish embryos when compared with the untreated control. However, the positive control showed abnormal morphological features, including yolk sac edema and bent spine (Figure 1).

#### 2.2.3. Effect of Peptide Toxicity on Zebrafish Larvae by Fluorescence Staining

**Figure 2:**
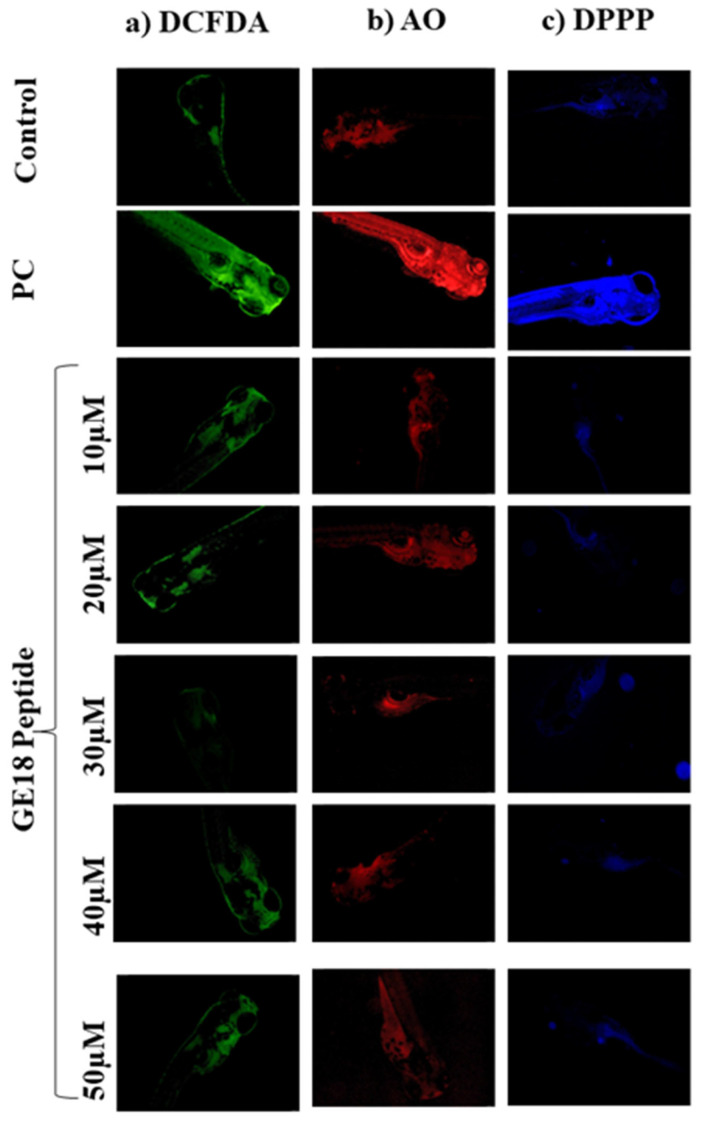
Fluorescent imaging assay on 96 hpf zebrafish larvae treated with GE18 peptide. Untreated was a control group, and positive control (PC) was treated with 1 mM of H_2_O_2_. (**a**) Intercellular ROS measurement by DCFDA, (**b**) apoptosis detected by acridine orange and (**c**) measurement of lipid peroxidation by 1,3-Bis(diphenylphosphino)propane (DPPP).

The outcomes of developmental toxicity data in the peptide challenge revealed that GE18 was non-toxic to the larvae. These results were further verified using a fluorescence staining approach by using DCFDA, AO and DPPP. Figure 2a shows the peptide-induced concentrations of ROS in the 96 hpf zebrafish embryos. The untreated control showed the minimum fluorescent intensity. The positive control (1 mM H_2_O_2_) showed the maximum fluorescent intensity, while the GE18 peptide-treated groups (10 to 50 µM) showed a slight variation in the fluorescent intensity. The ROS concentration in the control was 37.8± 1.5%, in the positive control was 84.53 ± 1.98% and the peptide-exposed groups were 37.46 ± 1.5% in 10 µM, 37.26 ± 1.36% in 20 µM, 39.59 ± 1.42% in 30 µM, 42.15 ± 1.85% in 40 µM and 43.1 ± 1.5% in 50 µM, in terms of fluorescent intensity (Figure S4A).

Figure 2 shows the observations of experiments where apoptosis was induced in 96 hpf zebrafish embryos in the presence or absence of GE18. The intensity of fluorescence in the peptide exposure groups (10 to 50 μM) was almost similar to the untreated control. However, the positive control group, as an indication of the occurrence of more apoptotic cells, showed a significant increase in the fluorescent intensity compared to the untreated control (Figure S4B). The fluorescent intensities in different groups were the control: 23.25 ± 1.5%; positive control: 93.25 ± 3%; peptide-exposed groups: 26.24 ± 1.5% in 10 µM, 24.25 ± 2.2% in 20 µM, 26.25 ± 1.8% in 30 µM, 30.94 ± 2% in 40 µM and 33.73 ± 1.5% in 50 µM.

The lipid peroxidation levels were evaluated through the DPPP staining assay. There was not much difference in the fluorescent intensities of the untreated control and the peptide-exposed groups. However, the positive control showed increased fluorescent intensity, indicating a higher lipid peroxidation level than the other groups (Figure 2c). Figure S4C represents the level of lipid peroxidation as an analysis of the fluorescent images on Image J software. The fluorescent intensities in the peptide-treated groups were 21.25 ± 1.65% in 10 µM, 22.32 ± 1.98% in 20 µM, 27.25 ± 1.32% in 30 µM, 33.25 ± 2.4% in 40 µM and 37.73 ± 2.36% in 50 µM. The positive control showed a significant increase in the fluorescent intensity at 87.7 ± 1.25% when compared with the control (19.25 ± 1.3%).

### 2.3. In Vitro Anti-Cancer Activity of GE18

#### 2.3.1. Anti-Proliferative Activity of GE18 on MCF-7 Cells

The MTT assay measured the anti-cancer effect of GE18. The results are shown in Table S2. The peptide exposure on MCF-7 cells led to the arrested cell proliferation in a dose- and time-dependent manner. The data, when compared with the control, showed a significant (*p* < 0.05) change. The IC50 values of the peptide on MCF-7 cells were 35.34 µM at 24 h and 29.06 µM at 48 h. The percentage of total LDH released from MCF-7 cells into the medium was measured. The results are shown in Figure S5. The peptide-exposed groups showed a dose-dependent increase in the concentration of the LDH level: 37.48 ± 2.3% in 10 µM, 43.67 ± 1.8% in 20 µM, 57.89 ± 3.54% in 30 µM, 66.89 ± 2.5% in 40 µM and 73.26 ± 1.62% in 50 µM. There was a significant (*p* < 0.05) difference between the outcomes of the control and the experimental groups.

#### 2.3.2. Morphology and Apoptosis

**Figure 3:**
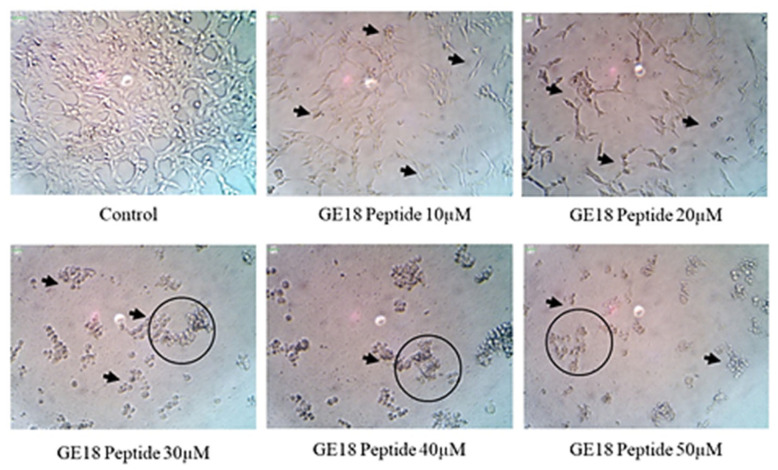
The morphological examination of GE18 peptide-treated cells at 20× magnifications in inverted phase-contrast microscopic view. The untreated cell was used as a control. Circles and arrows represented abnormal morphology.

The results from the morphological examinations are shown in Figure 3. These outcomes were compared with the untreated control group. While the control represented the physiological morphology of the MCF-7 cells, the peptide treatment groups showed abnormal morphological features such as detachment from the culture flask, loss of rigidness, rounding, shrinkage of cells and granulation of cells.

**Figure 4:**
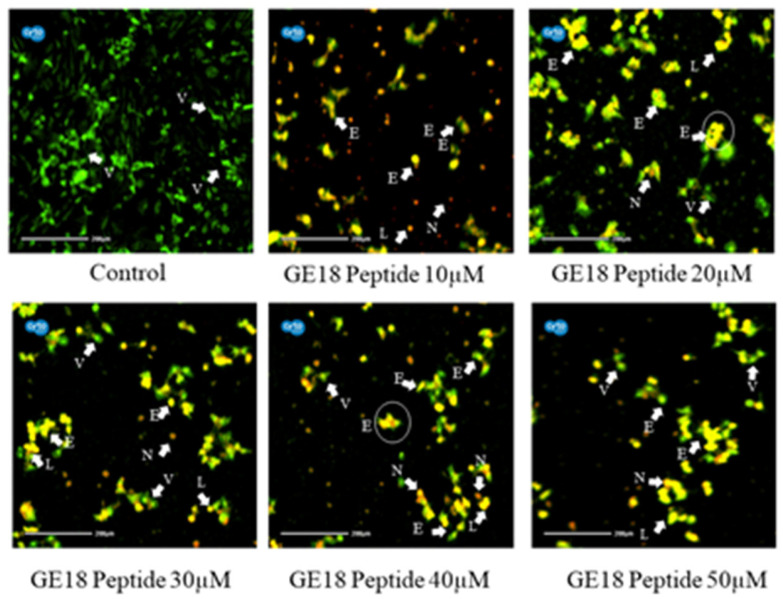
Photomicrographic representation of apoptotic stages by AO and EtBr staining on MCF-7 cells. Control (untreated) and GE18 peptide-treated cells. The stages were mentioned as V—viable cells; E—early apoptotic cells; L—late apoptotic cells; and N—necrotic cells. The white arrow indicates the apoptotic stages of cells.

Figure 4 reveals that the peptide-induced apoptotic cell death in breast cancer cells was more profound than the untreated control. Cell morphologies associated with early apoptotic, late apoptotic and necrotic cell death were observed in the peptide-exposed cells. Furthermore, different phases of apoptosis were also identified based on the emitted fluorescence during the AO/EtBr staining. Yellow indicates early apoptosis, reddish-brown indicates late apoptosis and brown indicates necrotic cell death; however, green represents viable cells.

#### 2.3.3. ROS Generation Potency

**Figure 5:**
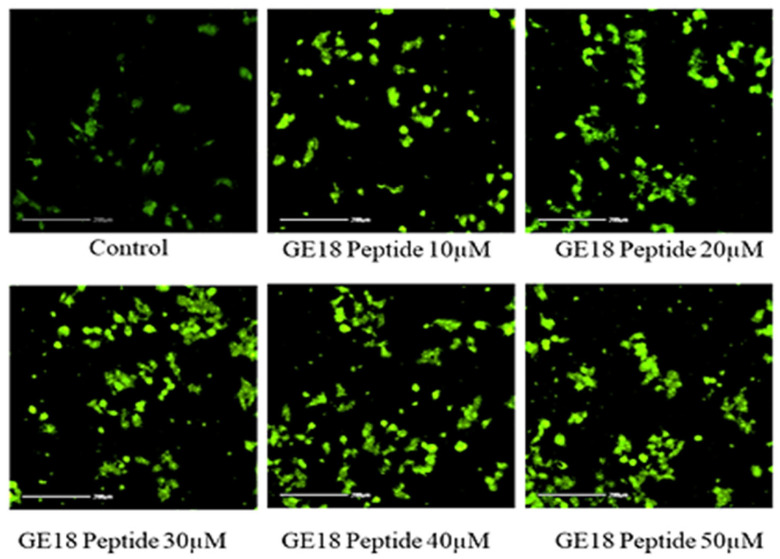
Intercellular ROS level measured by DCFDA. The GE18 peptide treatment generates ROS compared with the control (untreated) group.

The peptide-exposed MCF-7 cells showed a more elevated ROS concentration than the untreated control. Such an increased ROS concentration was dependent on the peptide concentration, i.e., GE18 dose-dependently elevated ROS concentration in breast cancer cells (Figure 5). The quantification data of DCFDA staining, as shown in Figure S6, demonstrated such a significant increase in ROS levels in the peptide-treated cancer cells, which is a concentration-dependent phenomenon.

#### 2.3.4. Mitochondrial Membrane Potential

**Figure 6:**
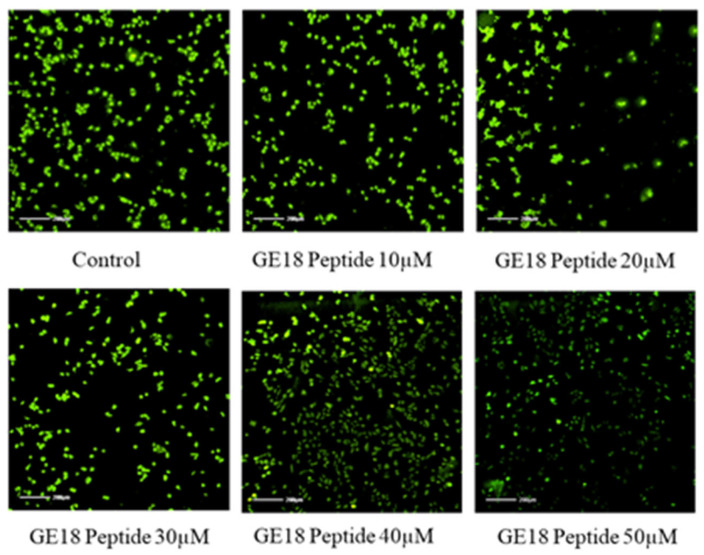
Mitochondria membrane potential activity by rhodamine 123; the peptide treatment group showed dose-dependent activity on MCF-7 cells compared with control (untreated) group.

The mitochondrial membrane potential of GE18 was analyzed by rhodamine 123 staining. The results showed that the peptide had dose-dependent activity on the mitochondrial membrane potential (Figure 6). This effect was further quantified by the fluorescent intensity using the ImageJ program. The peptide treatment groups showed a dose-dependent reduction in the fluorescent intensity (Figure S7). Compared with the untreated control, the peptide treatment groups showed significant activity.

#### 2.3.5. Scratch Assay

**Figure 7:**
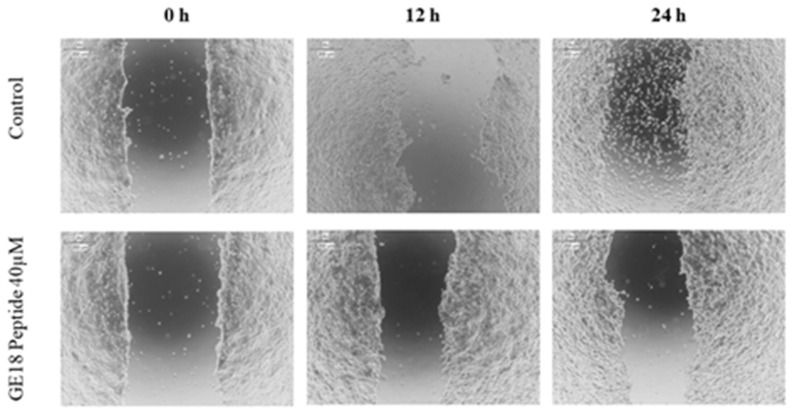
Scratch assay to determine the protective effect of GE18 peptides from cancer cell migration. The result was compared with the control (untreated) group.

The scratch assay results showed that the peptide had a protective effect in cancer cell migration; this was evaluated by the physically created wound in a monolayered cell culture. When compared with the untreated control, the peptide, at 40 µM concentration, arrested the cell migration in cancer cells, which is shown in Figure 7.

#### 2.3.6. qPCR Analysis

GE18 had the potential to regulate multiple anti-cancer genes in breast cancer cells. The peptide’s positive (up)regulation of genes were as follows: BAX: 2.87 ± 0.12-fold, Bcl-2: 0.34 ± 0.42-fold, p53: 4.35 ± 0.5-fold, Caspase-3: 2.98 ± 0.36-fold and Caspase-9: 6.25 ± 0.48-fold increase (Figure S8). Fold change values were calculated and normalized with the housekeeping gene GAPDH.

### 2.4. Evaluation of Anti-Microbial Activity of GE18

#### 2.4.1. MIC

A radial diffusion assay has been performed for ten different bacterial strains. The peptide GE18 showed significant activity against *P. aeruginosa,* as shown in Table 2. Hence, *P. aeruginosa* has been used for further anti-microbial analyses. To determine the minimum inhibitory concentration (MIC) of the GE18 peptide, the micro-dilution technique was performed using five different concentrations. The GE18 peptide treatment group showed bacterial inhibition percentages such as 35.68 ± 1.32% at 10 µM, 47.58 ± 1.45% at 20 µM, 59.24 ± 1.62% at 30 µM, 74.58 ± 1.23% at 40 µM and 83.26 ± 1.84% at 50 µM (Figure S9). However, the concentration above 60 μM showed 100% bacterial inhibition. GE18 peptide treatment showed concentration-dependent increased activity against *P. aeruginosa.* The MIC of GE18 peptide was found to be a 20 µM concentration. The results were compared with the untreated control, whereas the observed difference between the groups was found to be significant (*p* < 0.05). The concentration of GE18 was 40 µM, and 50 µM showed the maximum bactericidal activity.

#### 2.4.2. Anti-Bacterial Activity

The anti-bacterial activity of GE18 was established by the time kill assay, protein leakage assay and release of intercellular contents. The results, as shown in Figure S10, revealed that GE18 efficiently inhibited the proliferation of *P. aeruginosa*. Peptide exposure (40 µM, 4 h) arrested the cell proliferation, dose-dependently and time-dependently.

The peptide treatment damaged the *P*. *aeruginosa,* which resulted in the release of intracellular protein, as identified by the protein estimation assay. This peptide-induced effect was found to be a dose-dependent increase in the percentage of protein release. The concentrations of the released intracellular protein in the GE18-exposed groups were 29.48 ± 1.2% in 10 µM, 43.21 ± 1.35% in 20 µM, 56.89 ± 0.98% in 30 µM, 71.26 ± 0.78% in 40 µM and 83.26 ± 1.6% in 50 µM (Figure S11). The results, as compared with the untreated control, were significant (*p* < 0.05).

Observations from the release of the intracellular component from *P. aeruginosa* are shown in Figure S12. The peptide treatment groups showed a significant dose-dependent increase (*p* < 0.05) in the content release, when compared with the untreated control, which were as follows: 26.58 ± 1.2% in 10 µM, 39.85 ± 1.5% in 20 µM, 59.87 ± 1.74% in 30 µM, 77.63 ± 0.85% in 40 µM and 86.23 ± 1.36% in 50 µM.

#### 2.4.3. Inhibition of Biofilm Formation

Biofilms are exopolysaccharide matrices (EPM) generated by bacterial communities to attain multi-drug resistance properties. Biofilm inhibitory activity accessed anti-microbial peptides’ potency, calculating the minimum biofilm inhibitory concentration (MBIC). The GE18 peptide was tested against the pathogen *P. aeruginosa*, and the percentage of biofilm inhibitions was represented in Figure S13. The five different concentrations showed a dose-dependent increase in the inhibitory activity, which were as follows: 29.25 ± 1.6% in 10 µM, 41.3 ± 1.5% in 20 µM, 55.4 ± 1.74% in 30 µM, 69.83 ± 0.85% in 40 µM and 78.24 ± 1.36% in 50 µM. At the same time, the concentration above 60 μM showed 100% of the biofilm formation inhibition. The results were compared with the untreated control and positive control group. The MBIC was found to be 30 µM, which showed 55.4 ± 1.74% of biofilm inhibition.

#### 2.4.4. FE-SEM Analysis

**Figure 8:**
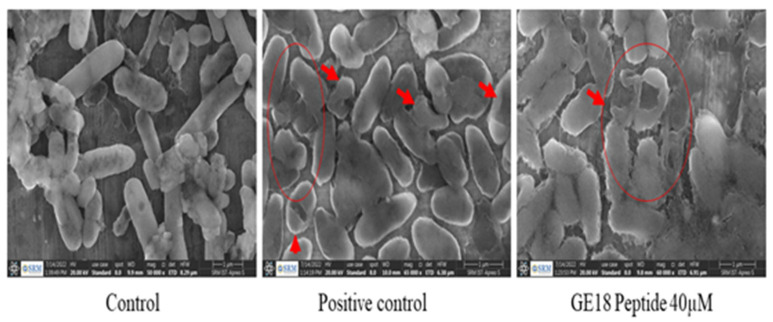
Effect of GE18 peptide influence on the morphological changes on *P. aeruginosa* by FESEM analysis. Control (untreated), positive control (1 mg/mL gentamicin) and GE18 peptide 40 µM. The morphological changes are represented in arrows and circles.

The effect of peptide on bringing out changes on the membrane surface and morphological changes was analyzed by the FE-SEM analysis. GE18 at 40 µM on *P. aeruginosa* significantly affected the cellular morphology, where shrinkage of the cell membrane and damaged cells were identified. The observed results were compared with the positive control (1 mg/mL gentamicin) and untreated control (Figure 8).

## 3. Materials and Methods

### 3.1. In Silico Analysis of Properties of the Peptide

From the previously reported transcriptomic data of *A. invadans*, the full-length cDNA sequence has been selected [12]. Then, the selected cDNA sequence was translated into the amino acid sequence of a protein using the online tool ExPASy (http://web.expasy.org/translate/ (23 November 2022) [14]. The converted protein sequence was subjected to a BLAST analysis. The Expasy’s ProtParam web server (http://us.expasy.org/tools/protparam.html (23 November 2022)) has been used to investigate the physiochemical properties of the protein [15]. The 3D structure of the protein has been predicted through the I-TASSER online platform and visualized in PyMol software (Version 0.99). The peptide sequence has been predicted through the HeliQuest online tool (http://heliquest.ipmc.cnrs.fr/cgi-bin/ComputParams.py (24 November 2022), and its physicochemical properties were analyzed as reported [16]. The ToxIBTL online tool (http://server.wei-group.net/ToxIBTL (24 November 2022) has been used to predict the toxicity of the peptide [17]. The identified peptide, GE18, has been synthesized by Zhengzhou Peptides Pharmaceutical Technology Co. Ltd., Zhengzhou, China, and the peptide’s purity was certified by the manufacturer using HPLC and mass spectrometry. A stock concentration of the peptide (1 mM) was prepared using phosphate buffer saline (PBS) maintained at −20 °C, and working concentrations were prepared at the time of the experiments.

### 3.2. GE18 Peptide Toxicity Analysis

#### 3.2.1. Maintenance of Cell Culture

The breast cancer cell line (MCF-7) and rat skeletal myoblast cell line (L6) were purchased from the National Centre for Cell Science (NCCS), Pune, India. Cells were maintained by continuous passaging in a medium supplemented with 10% fetal bovine serum (Gibco, Sydney, Australia), 1% of antibiotic-antimycotic solution (Sigma, St. Louis, MO, USA) and Dulbecco’s Modified Eagle Medium (DMEM) high glucose (4.5 g/L) (Sigma). The cells were kept in a 5% CO_2_ incubator at 37 °C.

#### 3.2.2. In Vitro Toxicity Assessment in Myoblast Cells

The peptide’s in vitro toxicity was analyzed by an MTT assay ((4,5-dimethylthiazol-2-yl)-2,5-diphenyltetrazolium bromide test) [18]. The L6 cells, maintained in a 5% CO_2_ incubator, were cultured on a 96-well plate for 24 h at 37 °C. Cells were seeded at a concentration of 5 × 10^4^/well into 96-well microplates. Cells were treated with the peptide (10 µL/well) at five different concentrations (10, 20, 30, 40 or 50 µM) for 24 h. Triton X-100 (10 µL/well)-treated cells were used as the positive control, and the untreated group was used as the control. Post-treatment, the medium was replaced, and the 20 μL MTT (5 mg/mL) solution was added to each well and incubated for 4 h. DMSO (200 µL) was used to dissolve the formazan crystals. Absorbance was measured at 570 nm using the ELISA reader, and the percentage of *cell viability* was calculated as follows.

$$\%\;cell\;viability=\left[\frac{OD\;of\;treated\;cells}{OD\;of\;untreated\;control}\right]\times 100$$

#### 3.2.3. In Vivo Toxicity Assessment in Zebrafish Embryos

Zebrafish (*Danio rerio*) were commercially purchased from the NSK aquarium, Kolathur, Tamil Nadu, India. Fish were acclimatized to laboratory conditions; for breeding and the collection of embryos, a standard protocol was followed [18,19,20,21]. The 4 h post-fertilized (hpf) embryos were examined under the microscope. Healthy embryos (30 per group) were selected, grouped into seven groups and maintained in the embryonic medium. The embryos, treated with hydrogen peroxide (1 mM, H_2_O_2_), served as a positive control. The untreated was considered as the control, whereas the experimental group was treated with five peptide concentrations (10, 20, 30, 40 or 50 µM). The treatment duration was 4 hpf to 96 hpf; parameters such as hatching rate, heart rate, survival rate, mortality rate and morphological abnormalities were analyzed.

### 3.3. Fluorescent Staining Assays

Assays were performed to determine the ROS concentrations, occurrence of apoptosis and lipid peroxidation levels. After 96 hpf, peptide-challenged, post-treated larvae from each group were utilized for the assays. Larvae were euthanized, fixed with 4% paraformaldehyde and then stained. Fixed larvae were stained with a 2,7-dichloro dihydro fluorescein diacetate (DCFDA) solution (20 µg/mL), Acridine orange (AO) (7 µg/mL) and 1,3-Bis(diphenylphosphino)propane (DPPP) (25 μg/mL) for 20 min [18,22,23]. After staining, the embryos were washed with PBS to remove the excess stain, and the photomicrographs were recorded in a fluorescence microscope equipped with a Cool SNAP-Pro color digital camera (Olympus, Tokyo, Japan). The fluorescence intensity was quantified using ImageJ software (V.1.49, NIH, Bethesda, MD, USA).

### 3.4. In Vitro Anti-Cancer Potency of GE18 Peptide

#### 3.4.1. Assessment of Anti-Proliferative Activity

The cancer cell proliferation inhibitory activity was calculated through the MTT assay on MCF-7 cells and the GE18 peptide (24 and 48 h exposure challenge) [24,25]. The standard protocol, as mentioned in Section 3.2.2, has been followed. The in vitro toxicity assessment and the percentage of *growth inhibition* were calculated as follows:

$$\%\;growth\;inhibition=\left[\frac{OD\;of\;untreated\;control-OD\;of\;treated\;cells}{OD\;of\;untreated\;control}\right]\times 100\%$$

The anti-cancer activity was measured by computing the IC50 value (the dose that induced 50% cellular damage) from GraphPad Prism Version 6 software’s inhibition rate data.

#### 3.4.2. Cytotoxicity Assessment by LDH Leakage Assay

The cytotoxicity of GE18 in MCF-7 cells was established through the LDH assay. Cells (5 × 10^4^/well) were treated with the peptide for 24 h at various concentrations (10, 20, 30, 40 or 50 µM). Untreated cells served as the control, while the Triton-X-100-treated cells served as the positive control. Post-treatment, cells were aspirated and centrifuged at 1200 rpm. A 50 μL sample was mixed with the 2 mL of the reaction mixture [NADH (0.20 mmol dm^−3^) and Tris buffer (61.43 mmol dm^−3^ pH 7.4)] and incubated for 15 min. Post-incubation, 200 μL of pyruvate (21.5 mmol dm^−3^) was added, and absorbance was measured at 339 nm using an ELISA reader. The percentage of LDH release was calculated [26,27].

#### 3.4.3. Morphological and Apoptosis Staining

The effect of GE18 on the cellular morphology was determined as described [28]. In brief, MCF-7 cells (4.0 × 10^5^/well) were grown on a six-well plate and treated with the peptide (10, 20, 30, 40 or 50 µM) for 24 h. Cells were examined using an inverted phase-contrast microscope. The results were acquired as pictures. Cells were double-stained using AO and ethidium bromide (EtBr) [29]. MCF-7 cells were grown on a six-well plate and exposed to the peptide (10, 20, 30, 40 or 50 µM) for 24 h. Cells were then stained with AO/EB (1:1 ratio; 10 µL of 100 µg/mL) for 30 min. Stained cells were washed with PBS, and then the apoptotic cells were identified by examination under a fluorescence microscope facilitated with a Cool SNAP-Pro color digital camera (Olympus, Tokyo, Japan). Results were recorded as photomicrographs.

#### 3.4.4. ROS Generation

The DCFDA assay measured the peptide-mediated influence on ROS generation in cancer cells [18,30,31]. In brief, the GE18 peptide-treated MCF-7 cells (4.0 × 10^5^/well) were stained by adding the 40 µM DCFDA solution for 20 min, and then the excess stain was washed with PBS and observed under a fluorescence microscope facilitated with a Cool SNAP-Pro color digital camera (Olympus, Tokyo, Japan). The results were recorded as photomicrographs. The fluorescent intensity of the images was measured by analyzing the images on ImageJ software (V.1.49, NIH, USA).

#### 3.4.5. Rhodamine 123 Staining

Whether or not GE18 influences the mitochondrial membrane potential was measured using the rhodamine assay [32]. In brief, cells were treated with the peptide for 24 h. Post-exposure cells (4.0 × 10^5^/well) were stained using rhodamine 123 dye (10 µg/mL) for 30 min in dark conditions. Then, the excess stain was washed with PBS, and the results were recorded as images by observing under the fluorescence microscope at 20× magnification. Fluorescent intensity was measured by processing the images in ImageJ software (V.1.49, NIH, USA).

#### 3.4.6. Cell Migration Assay

A wound-healing assay established the ability of GE18 to inhibit the migration of cancer cells [33]. Cells (4.0 × 10^5^/well) were grown as a monolayer on a six-well plate, and the scratch was made with a sterile plastic pipette tip (10 µL). Then, the monolayer cells were washed with PBS to remove the debris, and then the cells were challenged with the peptide. The results were recorded as images which were captured at 0, 12 and 24 h using a bright-field inverted microscope (40× magnification).

#### 3.4.7. In Vitro Anti-Cancer Gene Expression

The gene expression analysis has been executed to understand the molecular mechanism(s) by which GE18 elicits anti-cancer effects. A protein concentration in the range of 1 to 5 μg/μL of total protein in the cell lysate or tissue homogenate was required for the gene expression analysis. Using the Trizol method, total RNA was isolated from the MCF-7 cells (4.0 × 10^5^/well), which were treated with the peptide for 24 h. tRNA was converted into cDNA according to the ready script cDNA synthesis kit (Sigma). SYBR Premix ExTaq (Takara, Dalian, China) and Light Cycler 96 (Roche Applied Science, Basel, Switzerland) were used to perform the gene expression. The primers used are listed in Table 1, and GAPDH was used as the reference gene [34]. Results for the gene expression analysis are expressed in fold change values, as calculated by the 2^−ΔΔct^ method.

### 3.5. Anti-Microbial Potential of GE18

#### 3.5.1. MIC Radial Diffusion Assay

The radial diffusion assay has been performed to conduct a preliminary screen of the peptide for antibacterial activity against multiple bacteria [35]. A total of ten bacterial strains were tested, as mentioned in Table 2. With the help of a sterile swab, a bacterial lawn was prepared, and 2.5 mm diameter wells were punched in the agar. GE18 peptide (10, 20, 30, 40 or 50 µM) was loaded onto each well and kept for incubation. The zones of inhibition were measured. According to the results of the radial diffusion assay, further experiments were carried out using *P. aeruginosa*. To determine the minimum inhibitory concentration (MIC), a broth micro-dilution assay was performed [36]. Briefly, the 16 h log phase culture of *P. aeruginosa* was centrifuged. The cell pellet, which was resuspended in double-strength nutrient broth, was then challenged with the peptide at five concentrations. After the 3 h incubation, absorbance readings were taken at 625 nm in an ELISA reader.

#### 3.5.2. Time Kill Kinetics Assay

The bactericidal effect of GE18 has been determined by the time kill assay [36]. The mid-log phase culture of *P. aeruginosa* was challenged with the peptide in five concentrations, while untreated bacteria served as the control. The culture was withdrawn at various time points (0, 1, 2, 3, 4, 5 and 6 h) and plated on a nutrient agar plate, followed by incubation at 37 °C. The number of colonies formed was calculated at log10 CFU/mL to measure the inhibitory efficiency of the peptide.

#### 3.5.3. Protein Leakage Assay and Release of Intercellular Components

A protein leakage assay was performed with slight modifications [37]. For the experimental group, *P. aeruginosa* cells were treated with GE18 (10, 20, 30, 40 or 50 µM); untreated cells served as the control, and the gentamicin (1 mg/mL)-treated group served as the positive control. After the challenge, cells were centrifuged at 6000 rpm for 15 min. The supernatant was collected and utilized to quantify the protein concentration by the Bradford assay. The concentration of intercellular components that were released was measured by following Chen et al. and taking the absorbance values at 290 nm using an ELISA reader [38].

#### 3.5.4. Biofilm Inhibition Assay

The biofilm inhibition assay measured the peptide’s efficiency to prevent biofilm maturation [36]. Briefly, the log phase *P. aeruginosa* culture, at a concentration of 1 × 10^8^ cells/mL, was inoculated in a 96-well plate with a 200 μL culture volume. Then, the culture was exposed to the peptide at five different concentrations and incubated for 24 h at 37 °C. The untreated culture was considered as the control, and the gentamicin (1 mg/mL)-exposed group served as the positive control. Post-incubation, bacterial growth was measured. The biofilm formed was fixed with methanol stained with crystal violet, followed by washing the samples dry. Measurements were read at 570 nm.

#### 3.5.5. Morphology Analysis

The effect of GE18 on bacterial cellular morphology was examined through a field-emission scanning electron microscopy (FESEM) analysis [36]. The log phase culture was treated with GE18 for 2 h, the untreated group served as the control and 1 mg/mL of the gentamicin treatment group was considered the positive control. After treatment, the cells were fixed with 2.5% glutaraldehyde for an hour, which was followed by a gradient exposure with ethanol to dehydrate the sample (10% to 100% ethanol), and then the samples were air dried, and observed under FESEM. The results were recorded as images.

#### 3.5.6. Statistical Study

Data represent an average of three replicates ± standard deviation (SD). A one-way ANOVA and Tukey’s multiple range test using Graph Pad Prism (Ver.5.0) were applied to determine the statistical significance at the 5% level among all the performed assays.

## 4. Discussion

Different approaches have been tested and accepted as anti-cancer therapy. Yet, there is a need for efficient and precision target molecules with minimal adverse effects, for which a peptide-based treatment has been considered as an alternative in cancer therapy. Therapeutic peptides elicit anti-cancer effects by regulating multiple cellular mechanisms that target the cancer cells via tumor angiogenesis suppression, apoptosis, inhibition of distinct internal targets, membrane rupture and immune modulation [39,40]. AMPs were initially identified by their role in the innate immunity of a wide range of bacterial strains, which attracted the research community’s attention [41]. Different physicochemical factors influencing the effect of AMPs against cancer cells remain uncertain, although the properties of AMPs and ACPs are almost identical. Efforts are being undertaken to identify these similarities and differences, allowing for better ACP design [42]. Toxic molecules such as the venom of snakes, bees, scorpions, animal and plant pathogenic fungal-derived virulence factors have demonstrated potential anti-cancer activity [42,43,44,45], which is the fundamental supporting evidence for our proposition.

This study has investigated the anti-microbial and anti-cancer activity of the GE18 peptide, which is derived from the subtilisin-like peptidase or serine protease virulence factor of *Aphanomyces invadans.* The protein of interest has been identified from the cDNA sequence of *Aphanomyces invadans* transcriptome, and the protein has been named on the basis of the conserved domain present in the proteomic sequence. Similarly, the protein named subtilisin-like peptidase from the pathogen *Pseudogymnoascus destructans* functions as a virulence factor that causes white-nose syndrome in bats [46]. The protein subtilisin-like peptidase is the virulence factor that causes EUS in catfish. Hence, the selected protein sequence has been further analyzed to identify a bioactive peptide. The peptide, GE18, has been recognized as a lead molecule from the in silico analyses, (Table S1). The peptide properties matched the characteristic features of ACP and AMP as described previously [47,48].

GE18 originated from the virulent factor protein sequence, which should not be toxic to normal cells. Yet, its toxic effects were assessed in both in vitro (L6 cells) and in vivo zebrafish embryo models. The in vitro results showed that the GE18 is non-toxic, which was further established via the developmental toxicity assay on the zebrafish larvae. Zebrafish have several significant advantages over the other in vivo models [49,50,51]. The in vivo zebrafish has been considered an interesting option for innovative drug pharmacological screening, including toxicity, functional mechanisms, chemical libraries and gene expression studies [52,53,54,55]. The developmental toxicity results in Figure 1 and Figure S3 show that GE18 treatment on zebrafish embryos from 4 hpf to 96 hpf did not cause any toxicity, and it did not affect the hatching rate, heart rate, mortality rate, survival rate and general morphology of the zebrafish larvae. However, the positive control (1 mM H_2_O_2_) showed several adverse effects. Further, the effect of GE18, on whether or not it influenced the physiological cellular events such as ROS, apoptosis and lipid peroxidation were assessed. The results, as in Figure 2 and Figure S4, demonstrate that GE18 was almost similar to the untreated control, with slight variations. These findings correlate with the MP12 peptide, which was synthesized from the virulence factor of *Aphanomyces invadans* and showed significant anti-cancer properties against the Hep-2 cells [13]. The comprehensive in vitro and in vivo toxicity analyses suggest that GE18 is a non-toxic molecule.

The in vitro anti-cancer effect of GE18 has been established in breast cancer cells (MCF-7). GE18 is a small molecular weight molecule, >3 kDa, and it has ample charged amino acid residues HIS 1, SER 2, THR 1, GLY 3 and uncharged residues ARG 1, GLU 1, ASP 1, which are the key contributors to GE18′s anti-cancer effect [48]. GE18 showed a dose-dependent increase in the inhibition of cell viability. In support of this finding, a previous report showed that cell viability has been shown to be dose-dependent by an ICD-85 peptide with a molecular weight of less than 3000 Da. Findings from the total LDH assay showed a dose-dependent increase in the LDH level concentration, demonstrating that GE18 damaged the cell membrane of MCF cells. The MTT assay results correlated with the LDH assay outcomes. Similarly, it has been reported that the Brevilaterin B peptide from *Brevibacillus laterosporus* promotes the increase of LDH in epidermal cancer [56], wherein the positively charged amino acids are known to eventually lead to cell membrane damage. Likewise, an earlier report showed the peptide’s (isolated from *Grapsus albacarinous*) anti-cancer activity against MCF-7 cells [57]. GE18 affects the cellular morphology, which triggers the cells to undergo apoptosis for biological cellular elimination (Figure 4). This observation correlates with the Nymphayol treatment on MCF-7 cells, which eventually promotes apoptosis [34].

GE18-exposed MCF-7 cells upregulated intracellular ROS concentrations. A similar outcome has been reported previously, with a dipeptide from *Callyspongia fstularis* (marine sponge) symbiont *Bacillus pumilus* AMK1 showing a significant increase in the level of ROS [58]. It is also important to identify the type of cellular target (mitochondrial, cytosolic or nuclear) of a peptide to reach its anti-cancer effect. Typically, excess ROS leads to mitochondrial damage. Siddiqui et al. have reported on the dose-dependent effect of multiwalled carbon nanotubes on the mitochondrial membrane potential [59]. Based on this report, we suggest that GE18 elicits mitochondrial damage. Findings from the migration assay revealed that GE18 exposure inhibited cancer cell migration. The outcome correlated with the synthetic peptide YY1-EZH2, which showed anti-cancer activity against MCF-7 cells [60]. Anti-tumor medication therapy usually elevates ROS, which causes DNA damage and death by decreasing the mitochondrial membrane potential, resulting in the release of cytochrome C, activation of caspase3, increased BAX expression and reduced Bcl2 expression [61]. The gene expression analysis has identified the peptide’s functional role in activating and upregulating the four apoptotic genes, BAX, p53, caspase-3 and caspase-9. Similar results have been reported with VS-9, a peptide from *Allium sativum*, which induced caspase-3- and caspase-9-mediated apoptosis against leukemic cell lines [62].

The anti-bacterial activity of GE18 has been assessed against ten bacteria as a preliminary screening approach. Significant anti-bacterial activity was found against *P. aeruginosa* in the radial diffusion and MIC assays. This anti-microbial effect of GE18 is similar to the effect of RM12, a peptide from the Tachykinin protein derivative of *Channa striatus* [36]. The bactericidal effect of peptides may be promoted by the bacterial membrane damage caused by cell membrane integrity, structural disturbance, transport into the cytoplasm and adhesion to intercellular receptor molecules [63]. The peptide RM12 directly affected the bacterial membrane, and its anti-bacterial efficacy improved further due to its positively charged amino acid residues. Similarly, GE18 had positively charged residues that targeted the bacterial membrane, resulting in the loss of cell count; findings from the time kill assay showed a significant dose-dependent and time-dependent activity against *P. aeruginosa.* The protein leakage assay (Figure S11) and release of intercellular components assay (Figure S12) re-established the same outcome, as the peptide targeted the bacterial cell membrane for extensive damage, which led to increased protein centration and more release of the bacterial intercellular components to the culture medium. GE18 showed dose-dependent activity on the inhibition of biofilm formation by *P. aeruginosa*, which is in line with the outcome of the RM12 peptide [36]. The FESEM analysis showed the effect of GE18 to significantly damage the cell membrane. Similar to this effect by GE18, RM12 peptide has been reported to damage the cell membrane of *P. aeruginosa* [36].

## 5. Conclusions

Based on the outcomes of the study, we conclude that GE18, derived from the subtilisin-like peptidase or serine protease virulence factor of *A. invadans*, is non-toxic. Furthermore, the peptide has significant anti-cancer effects against breast cancer cells and anti-microbial activity against *P. aeruginosa*. Therefore, we propose that the peptide can be utilized for identifying therapeutical strategies against breast cancer or bacterial infection.

## Figures and Tables

**Table 1:** In vitro anti-cancer gene expression study list of primers used for the study.

Gene	Primer Sequence	Reference
*Bcl-2*	Forward: 5′-GTGGATGACTGAGTACCT-3′ Reverse: 5′-CCAGGAGAAATCAAACAGAG-3′	[34]
*BAX*	Forward: 5′-TCAGGATGCGTCCACCAAGAAG-3′ Reverse: 5′-TGTGTCCACGGCGGCAATCATC-3′	
*p53*	Forward: 5′-CCTCAGCATCTTATCCGAGTGG-3′ Reverse: 5′-TGGATGGTGGTACAGTCAGAGC-3′	
*Caspase-3*	Forward: 5′-ACATGGAAGCGAATCAATGGACTC-3′ Reverse: 5′-AAGGACTCAAATTCTGTTGCCACC-3′	
*Caspase-9*	Forward: 5′-GCTCTTCCTTTGTTCATC- 3′ Reverse: 5′-CTCTTCCTCCACTGTTCA-3′	
GAPDH (internal control)	Forward: 5′-GTCTCCTCTGACTTCAACAGCG-3′ Reverse: 5′-ACCACCCTGTTGCTGTAGCCAA-3′	

**Table 2:** List of bacterial strains used for preliminary screening of the anti-microbial activity of GE18 peptide. The antibacterial activity results were mentioned as susceptible (++++), intermediate (+, ++ or +++) and resistance (−).

Microorganism	Activity
*Aeromonas hydrophila* MTCC 1739	−
*Bacillus cereus* ATCC 2106	+
*B*. *subtilis* ATCC 6051	+
*Escherichia coli* ATCC 9637	−
*Klebsiella pneumoniae* ATCC 27736	−
*Pseudomonas aeruginosa* ATCC 25668	++++
*Staphylococcus aureus* ATCC 33592	++
*Serratia marcescens* MTCC 3124	−
*Salmonella enterica* MTCC 1166	−
*Vibrio harvey* MTCC 7954	+

## Data Availability

Data will be made available on reasonable request.

## Supplementary Materials

The following supporting information can be downloaded at: https://www.mdpi.com/article/10.3390/molecules28186746/s1.
