# Supplementary material for: Aquatic Peptide: The Potential Anti-Cancer and Anti-Microbial Activity of GE18 Derived from Pathogenic Fungus Aphanomyces invadans
Source: Molecules. 2023 Sep 21;28(18):6746. doi: 10.3390/molecules28186746 (PMC10534430; doi:10.3390/molecules28186746)
Supplement: Supplementary file 1 [file molecules-28-06746-s001.zip › molecules-2573816-supplementary.pdf]

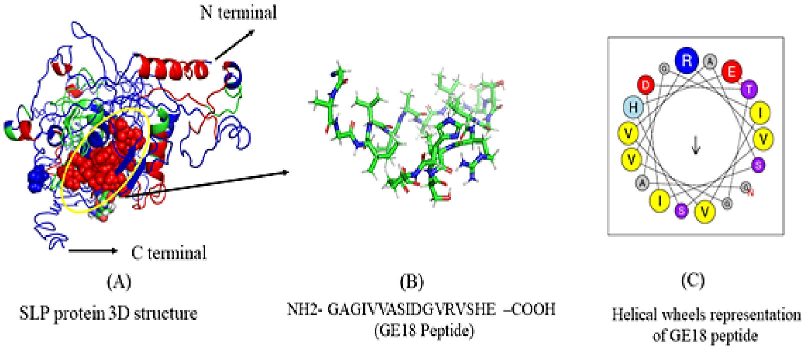

Figure S1. Bioinformatic analysis of Subtilisin-like peptidase protein (SLP protein) and GE18 peptide

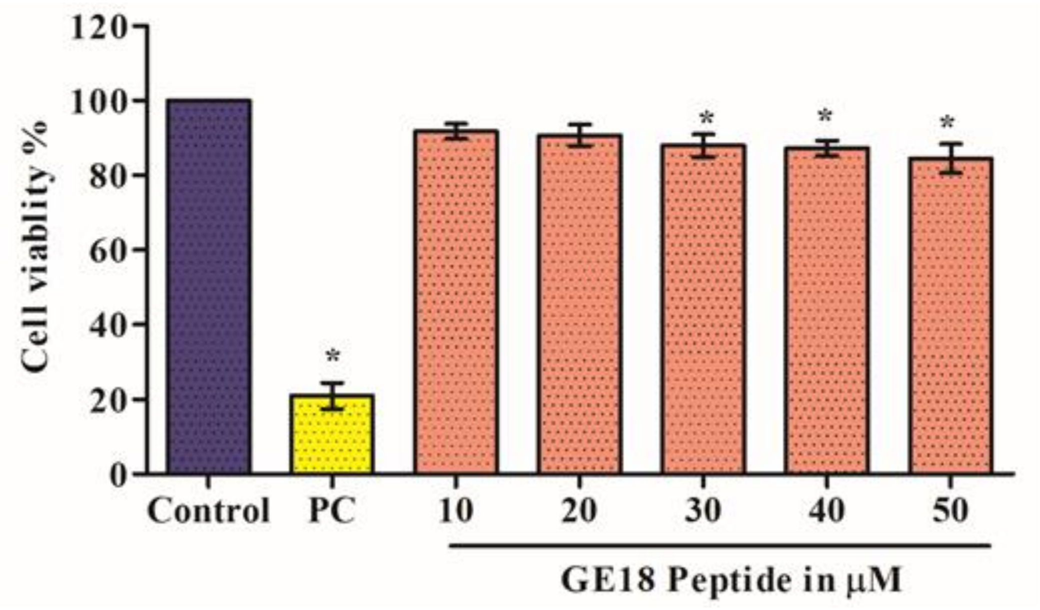

Figure S2. *In-vitro* toxicity assessment of GE18 peptide tested on L6 cell by MTT assay. GE18 peptide (five different concentrations), control (Untreated) group and positive control (PC) - Triton x-100 (0.01%). The ***** represented the level of significance ($p < 0.05$) when the results were compared to the control. Data were presented as mean ± SD for three independent experiments.

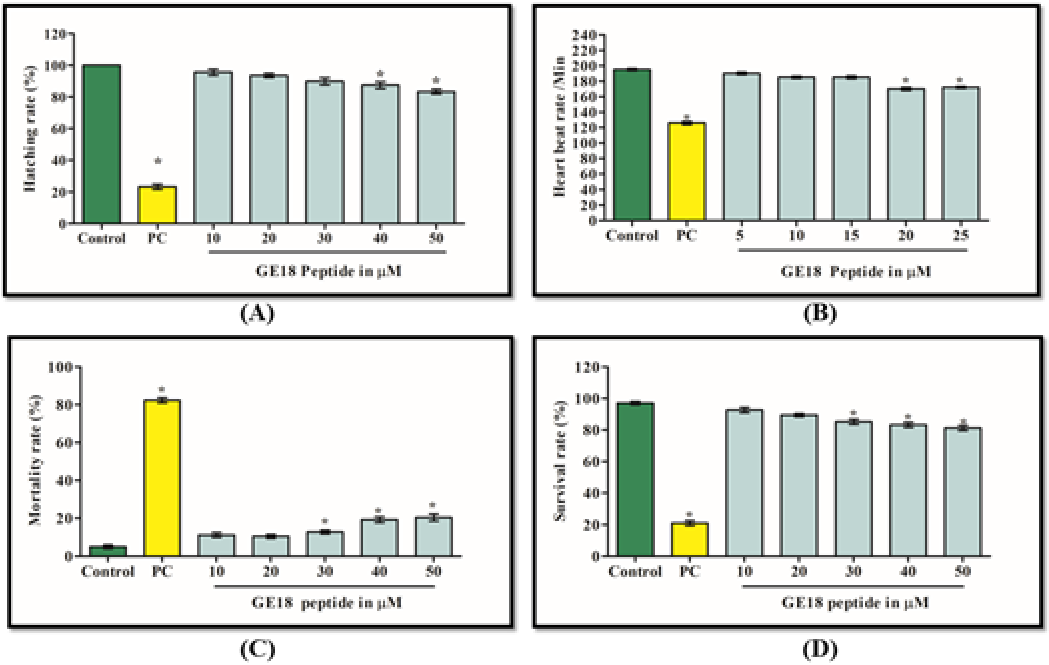

Figure S3. *In-vivo* zebrafish larva developmental toxicity study to determine the toxicity of GE18 peptide, Control (untreated group) and positive control (PC) - 1 mM of H_2O_2. (A) The hatching rate was calculated at 48 hpf, (B) The heart rate was calculated at 72 hpf, (C) The mortality rate was calculated at 96 hpf, and (D) The survival rate was calculated at 96 hpf. The results were compared with the control group, and statistical significance was represented by ***** $p < 0.05$, Data was mentioned as mean ± SD of three independent experiments

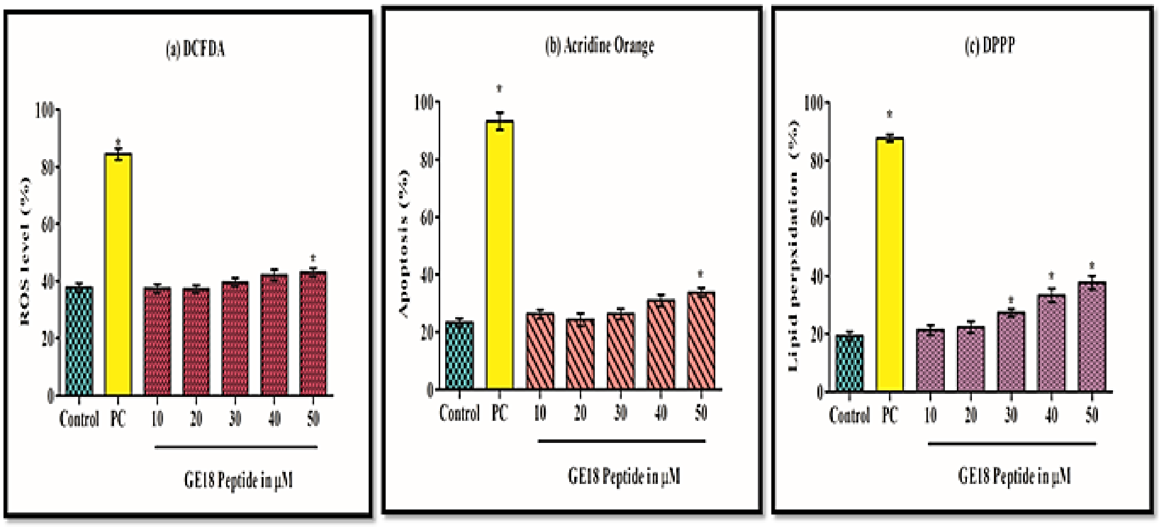

Figure S4. the fluorescent intensity measurement, images processed in Image J software. **(a)** DCFDA staining, (b) acridine orange-stating assay, and (c) DPPP assay. Experiments were performed in triplicate, and the data were expressed as mean ± SD. The asterix (*****) represents the statistical significance at $p < 0.05$ compared to the control

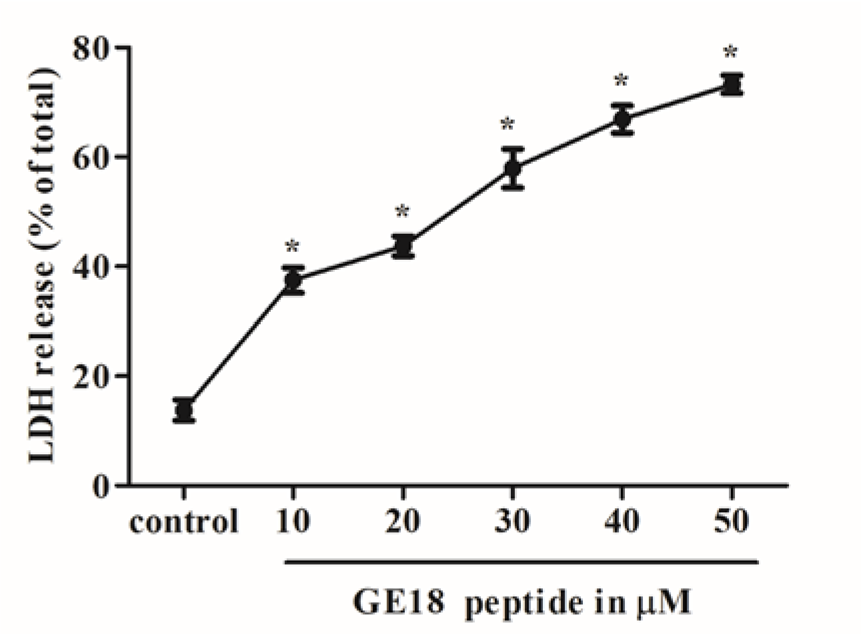

Figure S5. The total LDH release due to the effect of GE18 peptide on MCF-7 cells. The data of the peptide-treated group was compared with the control (Untreated) group and showed a statical significance of $p < 0.05$, indicated by the asterisk (*****), Data expressed as the mean ± SD of three independent experiments.

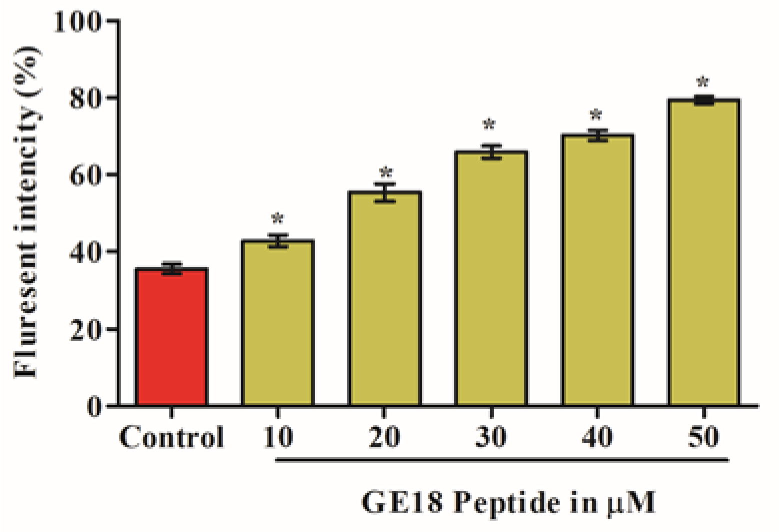

Figure S6. Quantitative measurement of the fluorescent intensity of DCFDA staining images processed in Image J software. Experiments were performed in triplicate, and the data were expressed as mean ± SD. The asterix (*) represents the statistical significance at $p < 0.05$ compared to the control.

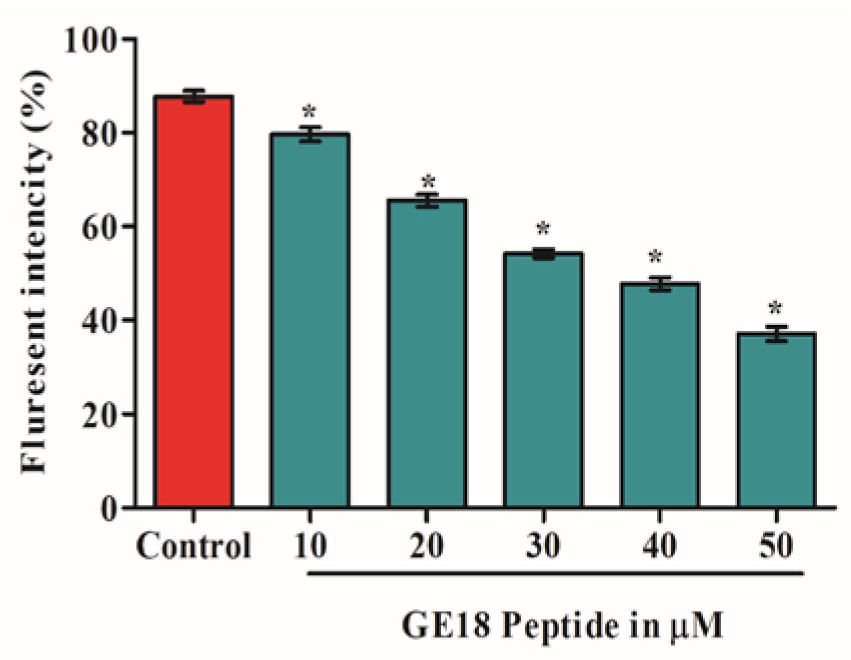

Figure S7. Quantitative measurement of the fluorescent intensity of rhodamine 123 staining images processed in Image J software. Experiments were performed in triplicate, and the data were expressed as mean ± SD. The asterix (*) represents the statistical significance at $p < 0.05$ compared to the control.

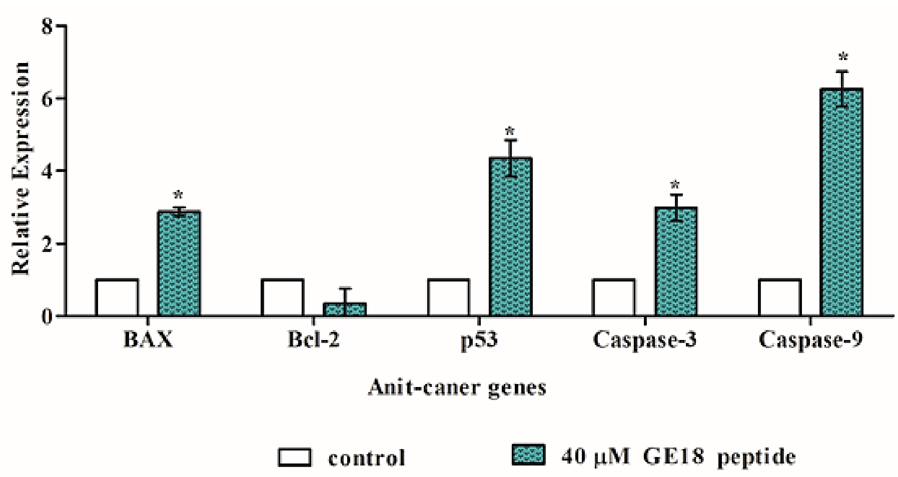

Figure S8. Anti-cancer gene expression analysis of GE18 peptide effect on MCF-7 cells, Genes expression calculations were done the data was normalised with GAPDH. The data were expressed as mean ± standard deviation (SD) of three independent experiments. The asterisk (***)** denotes the significance level at $p < 0.05$ compared to the control

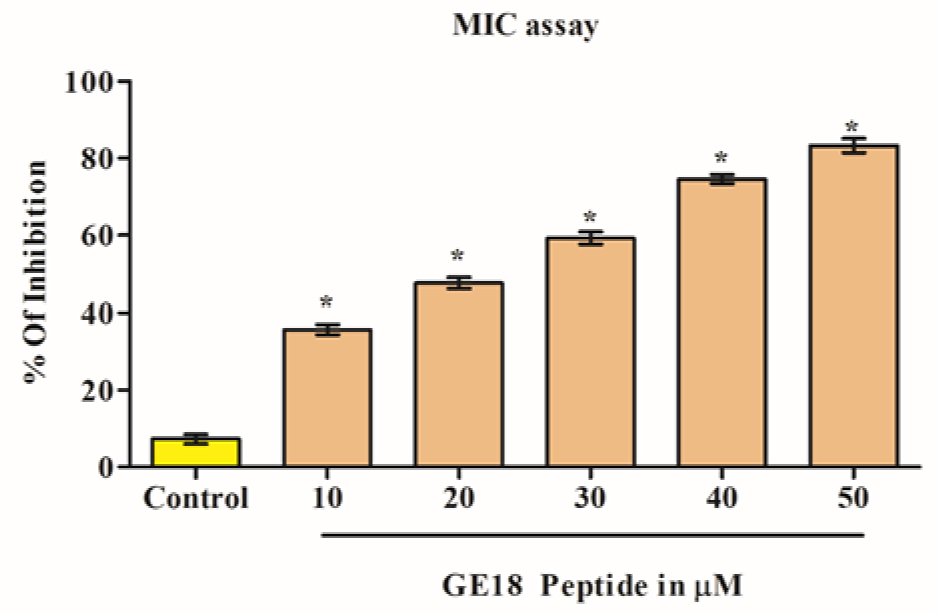

Figure S9. Determination MIC of GE18 peptide on *P. aeruginosa*, data were compared with the control group and showed a significance level of $p < 0.05$. data were represented as mean ± SD of three independent experiments.

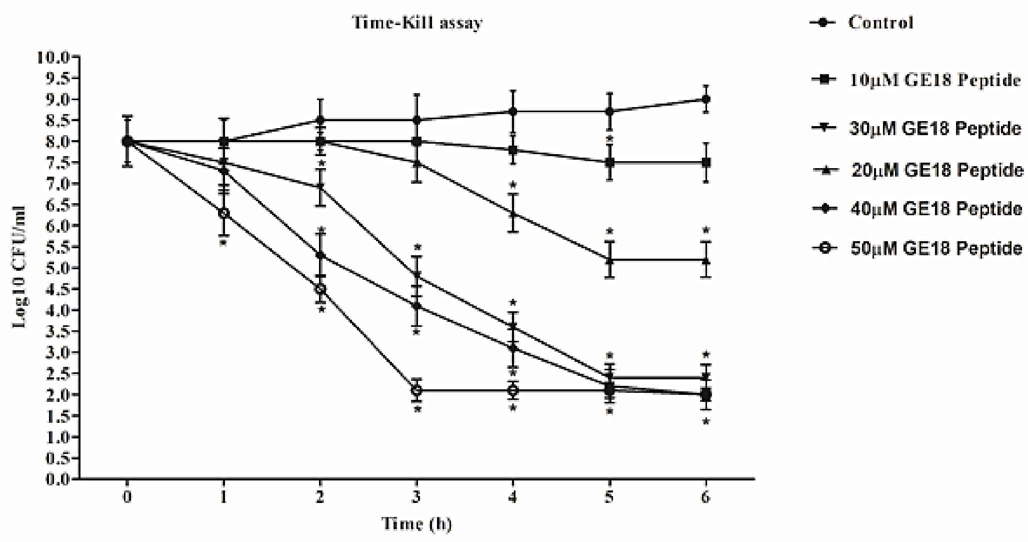

Figure S10. Determination of the bactericidal effect of GE18 peptide on *P. aeruginosa* by time-killing assay, control untreated and positive control (PC) 1mg/ml gentamicin. Data were compared with the control group and showed a $p < 0.05$. data were represented as mean ± SD of three independent experiments.

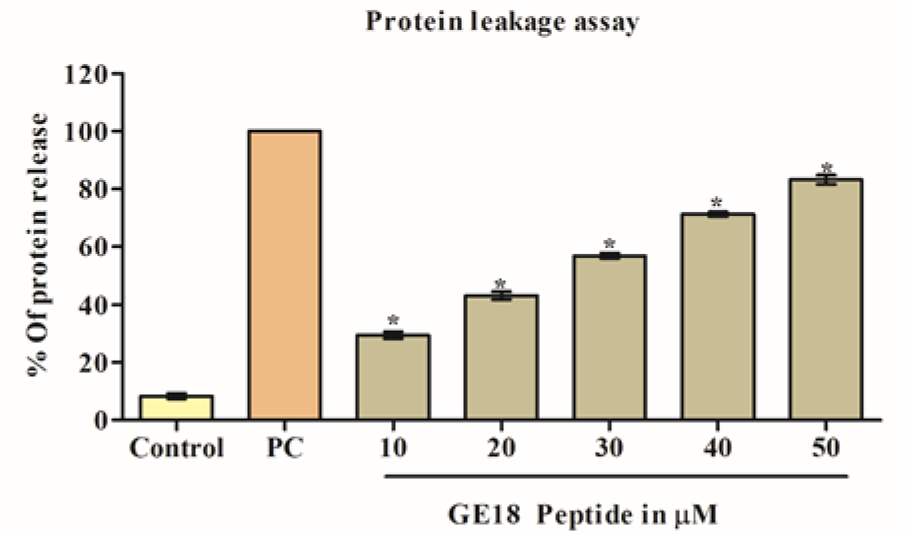

Figure S11. Determination of the bactericidal effect of GE18 peptide on *P. aeruginosa* by protein leakage assay, control untreated and positive control (PC) 1mg/ml gentamicin. Data were compared with the control group and showed a $p < 0.05$. data were represented as mean ± SD of three independent experiments

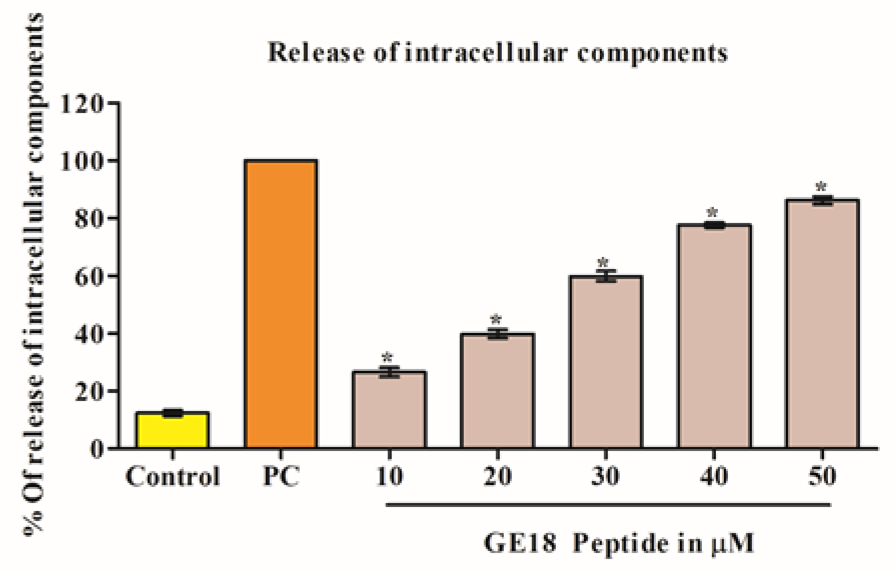

Figure S12. Determination of the bactericidal effect of GE18 peptide on *P. aeruginosa* by releasing intercellular components, control untreated and positive control (PC) 1mg/ml gentamicin. Data were compared with the control group and showed a $p < 0.05$. data were represented as mean ± SD of three independent experiments.

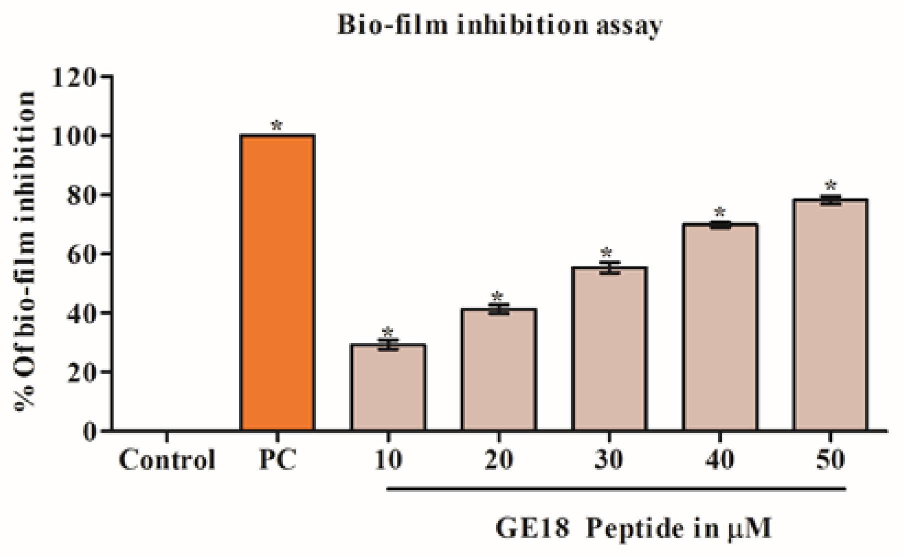

Figure S13. Determination of Bio-film inhibitory effect of GE18 peptide on *P. aeruginosa*, control untreated and positive control (PC) 1mg/ml gentamicin. Data were compared with the control group and showed a $p < 0.05$. data were represented as mean ± SD of three independent experiments.

Table S1. Phytochemical properties of Subtilisin-like peptidase protein and GE18 peptide analysed on Expasy's ProtParam tool.

**Properties**	Subtilisin-like peptidase (Protein)	GE18 Peptide (Peptide)
Number of amino acids	641	18
Molecular weight	67.16kDa	1.7 kDa
Theoretical pI	6.23	5.32
A total number of negatively charged residues (Asp + Glu):	45	2
A total number of positively charged residues (Arg + Lys):	39	1
Formula	$C_{2923}H_{4511}N_{833}O_{920}S_{35}$	$C_{75}H_{127}N_{23}O_{26}$
Total number of atoms	9222	251
The estimated half-life	>30 hours (mammalian reticulocytes, in vitro).<br>>20 hours (yeast, in vivo).<br>>10 hours (Escherichia coli, in vivo).	>30 hours (mammalian reticulocytes, in vitro).<br>>20 hours (yeast, in vivo).<br>>10 hours (Escherichia coli, in vivo).
The instability index (II)	32.69 This classifies the protein as stable.	-17.77 This classifies the protein as stable.
Aliphatic index:	69.13	118.89
Grand average of hydropathicity (GRAVY):	-0.09	0.622

Table S2. Cytotoxicity assessment to determine the anticancer potency of GE18 peptide, the results were compared with the control and the data were expressed as mean ± SD. The asterix (*****) represents the statistical significance at $p < 0.05$.

Concentration	24h	48h
**Control**	5.2 ± 0.25	7 ±0.35
**10μM GE18 Peptide**	26.57 ± 2.32*	25.23 ± 1.5*
**20μM GE18 Peptide**	33.59 ± 1.89*	39.25 ± 0.92*
**30μM GE18 Peptide**	42.58 ± 2.4*	47.85 ± 3.24*
**40μM GE18 Peptide**	53.21 ± 2.1*	58.6 ± 1.7*
**50μM GE18 Peptide**	61.75 ± 1.7*	67.25 ±1.8*
